# Cellular Cosmetics: How Innate Lymphoid Cells Can Recontour the Tumour Microenvironment

**DOI:** 10.1002/eji.70041

**Published:** 2025-09-03

**Authors:** Nabina Pun, David R. Withers

**Affiliations:** ^1^ Centre for Immuno‐Oncology, Nuffield Department of Medicine University of Oxford Oxford UK

**Keywords:** cancer | cytokines | innate lymphoid cells | tumour microenvironment

## Abstract

The innate lymphoid cell (ILC) family includes natural killer (NK) cells, recognised for over 50 years, as well as several more recently identified populations. Over the past 15 years, ILCs have emerged as key orchestrators of tissue homeostasis and inflammation. To build upon the early promise of cancer immunotherapies, it is essential to better understand the pathways regulating the composition of, and immunosuppressive mechanisms that dominate many solid cancers and effectively curtail or block T cell responses. Given their residence within most tissues, how these cellular sentinels influence tumour development and progression remains an active area of both discovery and more translationally focused research. By defining precisely how different immunosuppressive pathways form, rationalised immunotherapy combinations can be devised to specifically target these. Current evidence indicates that for each ILC subset, both pro‐ and anti‐tumourigenic roles are possible, likely reflecting local cues within different tissues and contexts. Here, we seek to concisely review some of the prevailing data describing ILC contributions to tumour immunity and highlight some of the challenges that still exist in fully dissecting these mechanisms.

## Graphical abstract

Innate lymphoid cells (ILCs) orchestrate tissue homeostasis and inflammation. Here, we review current understanding of how different subsets of ILCs can contribute to both pro‐ and anti‐tumour immunity depending on the local context.

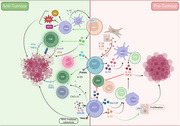

## 1 Introduction

Innate lymphoid cells (ILCs) constitute a functionally diverse collection of immune cells that develop from the common lymphoid progenitor (CLP) but lack the combinatorially rearranged receptors characteristic of B and T cells [1, 2, 3]. ILCs play a crucial role in orchestrating tissue homeostasis by responding to a diverse range of signals, including cytokines, leukotrienes, neurotrophic factors and microbial and dietary metabolites [4]. ILCs have been detected in many tissues but are particularly abundant at mucosal and epithelial barriers, where homeostasis is constantly challenged [2, 5]. Current nomenclature describes five subsets: natural killer (NK) cells, ILC1s, ILC2s, ILC3s and lymphoid tissue inducer (LTi) cells (Box 1) [4]. Most ILCs were first identified as early ‘innate’ sources of the effector cytokines characteristic of the different CD4^+^ T cell subsets [6, 7, 8, 9, 10]. Thus, in addition to maintaining tissue homeostasis, ILCs act as frontline responders during infection and inflammation, rapidly generating cytokines to expedite the immune response and likely compensating for the actions of effector T cells as these are expanded [1, 4, 5, 11].

Cancers commandeer tissues, disrupting normal processes to propagate their growth. A defining feature of the tumour microenvironment (TME) of solid cancers is the disruption of anti‐tumour T cell responses. Immunosuppressive mechanisms within the TME remain a major impediment to the efficacy of the breakthrough therapies of immune checkpoint blockade and CAR T cells [12, 13]. Understanding how the composition of the TME is established and sustained remains crucial to determining how best to shift its cellular makeup and promote anti‐tumour immunity. Given their ability to integrate and respond to local tissue signals, ILCs are perfectly equipped to influence the cellular balance and immune cell functions in the TME. Indeed, current evidence indicates that ILCs can either suppress or promote tumour development, depending on the context [14, 15]. Given recent NK cell‐focused reviews [16, 17, 18, 19], here we will consider the contributions other ILC subsets make in anti‐tumour immunity, concisely discussing the current understanding of their composition, mechanisms of action and the environmental cues that govern their behaviour and function.

## 2 Tissue‐Specific ILC Compartments

ILC subsets are unevenly distributed throughout the body. For example, ILC3s predominate in the small intestine, whereas ILC2s are more abundant in the lungs [4, 58]. Seminal parabiosis experiments established that ILC populations in organs, including the lung, small intestine and liver, are predominantly host‐derived, with over 95% remaining tissue‐resident even during systemic immune perturbations [59]. Thus, most ILCs appear to permanently reside within tissues, serving as sentinels that orchestrate local homeostasis.

Analogous to the seeding of tissue macrophages during embryonic haematopoiesis [60], waves of ILC progenitors colonise tissues both pre‐ and post‐natally, then differentiate to form the tissue‐specific ILC compartments [44, 61, 62, 63, 64]. Although this process is incompletely defined, ILC progenitor populations appear to be retained within tissues and support local expansion when required, rather than needing maintenance from bone marrow (BM)‐derived cells. Nevertheless, tissue ILC compartments can be replenished with BM‐derived progenitors, as demonstrated in the many BM chimaera experiments performed to date [65, 66, 67, 68, 69]. Consistent with these observations from mice, recent studies of human tissues indicate the presence of ILC progenitors and tissue‐specific transcriptional programmes for ILC subsets [36, 37, 61, 70, 71].

Although most reside in tissue, ILCs are present in the circulation and can be induced to disseminate from specific tissues to draining lymphoid tissue and beyond. Human peripheral blood has long been used as a source for studying ILCs [1, 11] and contains ILC progenitors alongside more mature populations [61, 70]. Photo‐labelling of murine lymph nodes (LNs) has demonstrated the recirculation of some ILCs, particularly ILC1s, through lymphoid tissues [72]. Site‐specific tissue labelling indicates that ILC3s can exit the small intestine and migrate to the draining mesenteric LNs during infection [73], whereas increased ILC numbers have also been described in tumour‐draining LNs in mouse models [74]. A particularly dynamic example of ILC trafficking is the interleukin (IL)‐25—driven dissemination of intestinal ‘inflammatory’ ILC2s, which enter circulation via sphingosine‐1‐phosphate—mediated chemotaxis and migrate to distant tissues such as the lungs, where they participate in defence and repair [41, 42, 43]. Of note, elegant recent studies have provided further direct evidence of ILC2 migration from the gut to the pancreas [75, 76]. Furthermore, such migration can be controlled by adrenergic neuronal signals, specifically through limiting gut ILC2 retention and thus supporting the egress of these cells to the pancreas to then influence glucose metabolism [76]. Collectively, these data argue that ILC migration between tissues occurs within certain contexts; however, the mechanisms regulating this behaviour remain incompletely described.

**BOX 1 Classification and functional diversity of innate lymphoid cells (ILCs)**

ILCs were initially classified into three main groups based largely on their cytokine secretion profiles [1, 2]. The nomenclature was updated in 2018 to reflect a better understanding of their development and function [4] and now describes five subsets: NK cells, ILC1s, ILC2s, ILC3s and Lymphoid Tissue Inducer (LTi) cells. The numbering of ILCs reflects the nature of the immune responses in which they chiefly participate. Type 1 immunity is designed to deal with intracellular challenges, both infectious, but also cellular transformation [20]. Type 2 immunity is crucial in the defence against parasites, tissue repair and wound healing [21, 22]. Type 3 immunity plays a critical role in maintaining epithelial barriers and responding to extracellular pathogens [23, 24].

**NK cells**: NK cells are highly cytotoxic lymphocytes whose functions are controlled by a wide array of germline‐encoded activating and inhibitory receptors [25, 26, 27]. They are key early producers of interferon (IFN)γ [28, 29], supporting the generation of responses against intracellular infections and cancerous cells. Of note, NK cell development depends on both T‐bet and eomesodermin (Eomes) [30].

**ILC1s**: ILC1s were initially identified as non‐ or weakly cytotoxic lymphocytes that readily produce the defining Type 1 cytokines IFNγ and tumour necrosis factor (TNF)α in response to intracellular infections [31, 32]. ILC1s can be distinguished from NK cells through their Eomes‐independent development [33]. Notably, cytotoxic ILC1 states have recently been described, with the transcription factor Hobit identified as a key mediator of ILC1 differentiation [34, 35]. Furthermore, it is now evident that tissue‐specific ILC1 compartments exist in both mice and humans [36, 37].

**ILC2s**: ILC2s are defined by their production of the Type 2 cytokines interleukin (IL)‐4, IL‐5 and IL‐13, alongside high expression of the transcription factor GATA‐3 [8, 9, 38]. ILC2s typically respond to IL‐25, IL‐33 and thymic stromal lymphopoietin (TSLP) to orchestrate helminth clearance and tissue repair, as well as drive allergic inflammation [39, 40]. Although ILC2s appear more homogeneous across tissues than other ILCs, IL‐25 stimulation induces a distinct ‘inflammatory’ subset that rapidly disseminates to enhance mucosal defence [41, 42, 43].

**ILC3s**: ILC3s are defined by their expression and dependency on the transcription factor retinoic acid receptor orphan receptor (ROR)γt, their post‐birth development and their expression of IL‐22, but also IL‐17 in some contexts [7, 44]. ILC3s play a crucial role in mucosal barrier maintenance [45, 46, 47, 48] as well as in the regulation of adaptive immune responses [49, 50, 51]. Multiple subsets of ILC3s are evident, underpinned by distinct transcriptional programmes [52], although their specific in vivo functional contributions remain incompletely resolved, largely due to the challenges of specifically targeting these subsets.

**LTi cells**: LTi cells were initially identified as CD4^+^CD3^−^ cells that support the formation of lymph nodes and Peyer's patches during embryonic development [53, 54]. Although LTi cells are dependent on RORγt, they have distinct developmental requirements compared to ILC3s [55, 56, 57] and to reduce terminology‐induced confusion, the LTi cell subset specifically describes the RORγt‐dependent compartment formed in the embryo. Given their development‐restricted role, these ILCs will not be further discussed here.

## 3 ILC Populations in Cancer

NK cells are prominent anti‐tumour effectors, recruited from circulation into cancers to target stressed or antigen‐deficient cells. However, within the TME, circulating NK cells can acquire transcriptional and phenotypic profiles resembling ILC1‐like cells [36, 77, 78].

By contrast, non‐NK ILC subsets in solid tumours remain under‐investigated, particularly regarding their origins, whether from circulation or local tissue. Given that most ILCs reside in tissue, one might anticipate that locally derived cells are most heavily involved, either as mature ILCs or at the stage of tissue‐resident progenitor populations. ILC plasticity may also complicate accurate identification of the source of specific populations [79]. Syngeneic cell line implantation, frequently performed subcutaneously, generates tumours that contain a clear NK cell compartment but few other ILCs [74, 78]. These rapidly growing tumours form between the skin and muscle layers, likely limiting the recruitment of local cells from tissue. Hence, the paucity of ILCs probably reflects the limited recruitment possible from the blood.

More physiologically relevant models, utilising organoid implantation [80] or spontaneous tumour development, are essential to understand intratumoural ILC composition. Of note, analysis of a spontaneous model of breast cancer identified distinct granzyme B and granzyme C‐expressing ILC1 cells that appear to be derived from a local tissue‐resident population within the mammary gland [81, 82]. Significant increases in the number of ILC1 and ILC3 in the colon tissue of azoxymethane (AOM)/dextran sodium sulphate (DSS)‐treated mice, a model of colitis‐associated cancer, have also been described [74]. These data indicate that the local tissue ILC compartment does influence the intratumoural ILC population. An important further consideration is the accurate identification of *bona fide* ILCs within tumours, often undermined by gating strategies and lineage ‘dump’ channels that don't consider down‐regulation of key molecules such as CD3 by intratumoural T cells.

Although ILC2 infiltration of primary pancreatic ductal adenocarcinoma (PDAC) has been reported and correlated with longer survival [83], for many cancer types, observations regarding changes in ILC compartments in cancer patients have largely focused on blood. Changes in circulating non‐NK ILC subsets have been reported in metastatic colorectal cancer (CRC), gastric cancer, non‐small cell lung cancer (NSCLC), chronic lymphocytic leukaemia (CLL) and acute myeloid leukaemia (AML) [84, 85, 86, 87, 88]. This might suggest disruption to local ILCs; however, these data are limited to differences in the proportions of ILCs within the CD45^+^ compartment or changes in the proportion of specific subsets within the ILC compartment. Given the persistence of most ILCs within tissues, precisely what these observations mean for ILC function in specific cancer contexts remains unclear.

## 4 Subset‐Specific Roles of ILCs in Tumour Immunity: Friend or Foe?

**FIGURE 1:**
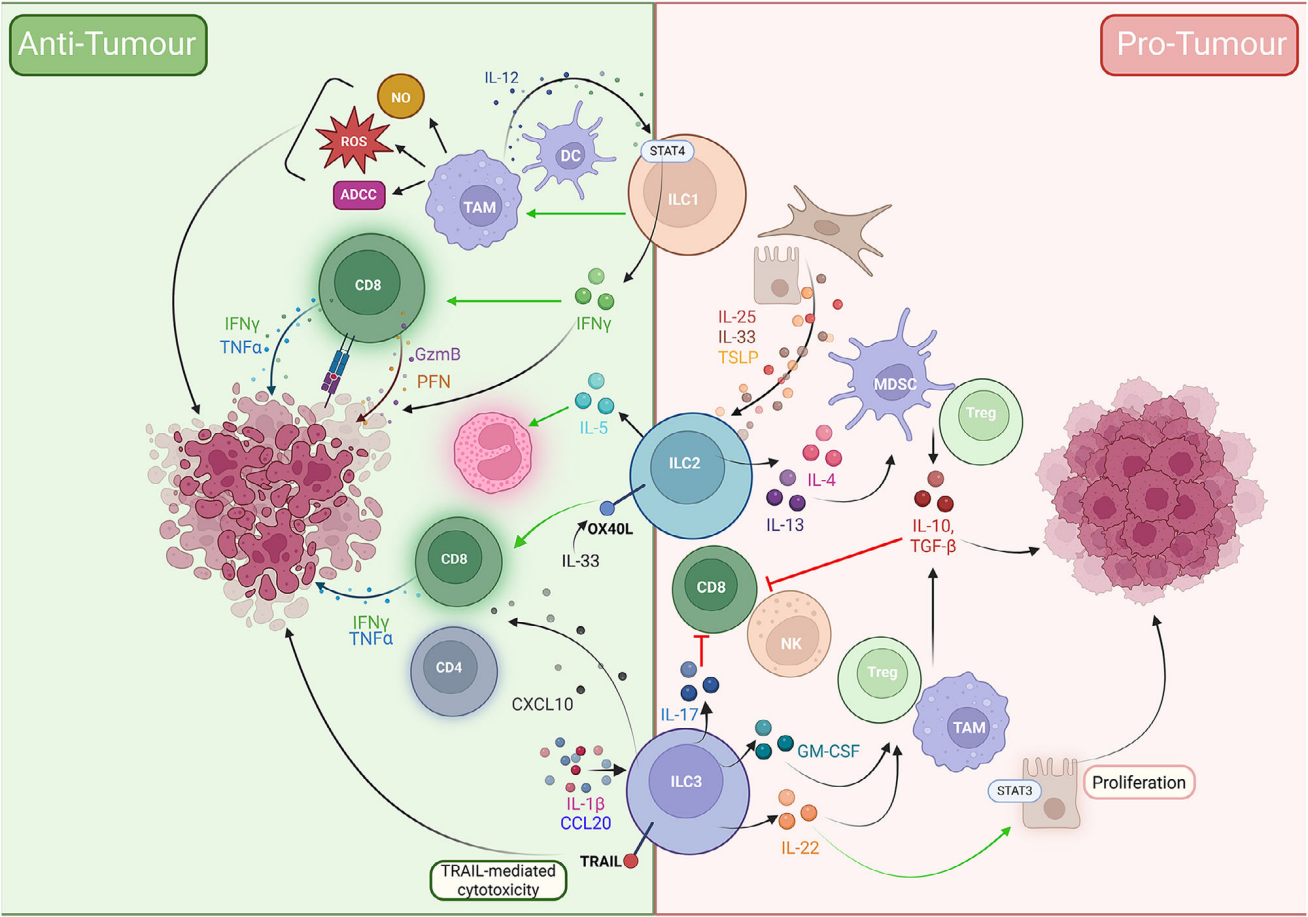
Pro‐ and anti‐tumour roles of ILCs in the TME. This schematic illustrates the dual roles of ILC subsets (ILC1, ILC2 and ILC3) in modulating anti‐tumour (left, green) and pro‐tumour (right, red) immunity within the TME. **Anti‐tumour functions**: ILC1s are activated by IL‐12 from dendritic cells (DCs) and tumour‐associated macrophages (TAMs), leading to signal transducer and activator of transcription (STAT)4‐dependent production of IFNγ. IFNγ promotes Type 1 immunity, enhances CD8⁺ T cell cytotoxicity and reprogrammes TAMs towards a pro‐inflammatory phenotype, increasing nitric oxide (NO), reactive oxygen species (ROS) and antibody‐dependent cellular cytotoxicity (ADCC). Tumour cells are also killed via granzyme (GzmB) and perforin (PFN) release. ILC2s can exert anti‐tumour effects by producing IL‐5 which activates and drives eosinophil recruitment. ILC2s also upregulate OX40L in response to IL‐33, thereby enhancing CD8⁺ T cell activation and the anti‐tumour response. In response to IL‐1β and CCL20, ILC3s can support anti‐tumour immunity by secreting CXCL10 (recruiting CD4⁺/CD8⁺ T cells) and, in some contexts, mediating direct TNF‐related apoptosis‐inducing ligand (TRAIL)‐dependent cytotoxicity against tumour cells. **Pro‐tumour functions**: ILC2s are activated by epithelial and cancer‐associated fibroblast‐derived alarmins (IL‐25, IL‐33 and thymic stromal lymphopoietin (TSLP)), producing IL‐4 and IL‐13, which recruit and activate myeloid‐derived suppressor cells (MDSCs) and regulatory T cells (Tregs). This results in the suppression of CD8⁺ T cell and NK cell cytotoxic functions. Recruitment of TAMs and Tregs further suppresses anti‐tumour immunity by producing IL‐10 and TGF‐β. ILC3s promote tumour progression via IL‐22 (activating STAT3 in epithelial cells to drive proliferation) and IL‐17 (suppressing CD8⁺ T cell responses). ILC3s also recruit Tregs and TAMs through GM‐CSF and support metastasis via RANKL induction. Green arrows represent activation. GM‐CSF, granulocyte‐macrophage colony‐stimulating factor; IFN, interferon; IL, interleukin; ILC, innate lymphoid cell; NK, natural killer; TME, tumour microenvironment; TNF, tumour necrosis factor. *Source*: Created in BioRender. Withers (2025) https://BioRender.com/onxgxnz.

Due to their strategic positioning in peripheral tissues, ILCs are well‐placed to sense and influence the TME. Their ability to rapidly respond to cytokines, alarmins and metabolic cues enables them to shape early immune responses. These roles are summarised in Figure 1.

***ILC1s***: ILC1s, traditionally considered less cytotoxic than NK cells but with robust cytokine‐producing functions, can contribute to anti‐tumour immunity through supporting dendritic cell (DC) activation and promoting T cell responses, particularly through interferon (IFN)γ production [81]. IL‐12, primarily produced by DC and macrophages within the TME [89, 90], activates ILC1s via signal transducer and activator of transcription (STAT)4 signalling, promoting cytokine release and Type 1 immunity‐focused effector T cells [91, 92]. IFNγ can also help reshape the myeloid compartment of tumours and push tumour‐associated macrophages (TAMs) towards a pro‐inflammatory phenotype that enhances anti‐tumour activity through nitric oxide production, reactive oxygen species and promotion of antibody‐dependent cellular cytotoxicity (ADCC) [93]. Thus, ILC1‐produced cytokines may help shift the balance of TAM composition, but data on this are currently more suggestive than conclusive [93].

Although currently only described in certain murine tumour models and tissues to date, ILC1 expression of granzymes supports a potential role in direct tumour cell killing and this ability has been demonstrated in vitro [81]. Tumours from patients with clear cell renal cell carcinoma (ccRCC) and chromophobe renal cell carcinoma (chR) contained an ILC1 population expressing granzyme A and were associated with better prognosis [94]. Interestingly, granzyme A expression by intratumoural ILC1 in chRCC appeared higher than in the surrounding kidney, suggestive of TME‐specific changes in these ILC1s [94]. This more cytotoxic ILC1 response appears to be driven by IL‐15 [82, 94].

**Summary/Perspective**: Through local production of Type 1 cytokines alongside emerging cytotoxic functions, ILC1s can clearly contribute to the anti‐tumour response in the TME, although functions may be overlapping with multiple other lymphocytes. Only a handful of differentially expressed genes between ILC1s and NK cells have been identified, with tissue‐adapted NK cells closely resembling ILC1s [71].

***ILC2s***: Type 2 responses are generally anti‐inflammatory and focus on minimising tissue damage by promoting macrophage‐mediated remodelling rather than aggressive pathogen elimination [95]. Tumours can exploit this wound healing‐like environment, hijacking immune pathways to create an immunosuppressive and pro‐angiogenic TME that favours their growth and survival [96, 97]. ILC2 activation is principally orchestrated by the alarmins IL‐33, IL‐25 and TSLP, which are released by epithelial cells under stress, damage or transformation [98, 99]. These alarmins have all been implicated in supporting tumour progression [99, 100, 101]. The response of ILC2s to these cues is the production of cytokines, including IL‐4, IL‐5, IL‐9 and IL‐13, alongside further mechanisms that collectively contribute to the recruitment and activation of immunosuppressive cells such as myeloid‐derived suppressor cells (MDSCs) and regulatory T cells (Tregs) [8, 9, 38, 96, 102]. For example, in a genetic mouse model of Apc mutation‐driven CRC, high IL‐25 expression (by tuft cells) and IL‐25R‐expressing tumour‐resident ILC2s are associated with impaired anti‐tumour responses and reduced survival [97]. Specifically, IL‐25 stimulation enhanced ILC2‐driven recruitment of MDSCs, which inhibit CD8^+^ T cell activity and accelerate tumour growth; blocking this IL‐25/ILC2 axis reverses immunosuppression and reduces tumour burden, highlighting its therapeutic potential [97]. Tuft cell and ILC2 gene signatures predict reduced survival in intestinal‐type gastric cancers, with 40% of these cancers displaying co‐enrichment of tuft cells and ILC2s [103]. Genetic depletion of tuft cells or ILC2s alleviates gastric metaplasia and reduces tumour burden in mouse models of gastric cancer [103]. The IL‐25/ILC2 axis has also been implicated in NSCLC, where high IL‐25 expression correlated with worse survival [101]. Adoptive transfer of IL‐25‐activated ILC2s in mice revealed increased tumour growth, metastasis and reduced survival through the accumulation of MDSCs in tumours [101]. In lung inflammation, ILC2 can dictate Treg suppressive function and local immunity [104, 105], suggesting this may be a key axis impacting anti‐tumour responses in the tissue; however, further investigation is needed to elucidate its function in the context of cancer.

Increased expression of IL‐33 has been reported in multiple cancers [106]. IL‐33‐induced ILC2 effector functions are regulated by peroxisome proliferator‐activated receptor gamma (PPARγ), a ligand for which is derived from prostaglandin D2 [107, 108]. IL‐33 promoted tumour growth in breast cancer by expanding IL‐13‐producing ILC2s, Tregs and MDSCs, while also reducing NK cell [100]. More recently, IL‐33‐induced activation of lung ILC2 was shown to impair NK cell function and increase tumour burden in a model of metastatic lung disease [109]. Specifically, IL‐5‐induced lung eosinophilia, downstream of ILC2 function, suppressed NK cell effector functions through disrupting normal metabolism [109]. These studies highlight the extensive crosstalk between ILC2 and other immune cells within the TME and how this can influence tumour control.

TSLP is upregulated in pancreatic tumours and is associated with a Th2‐dominant immune microenvironment, which correlates with worse patient outcomes [110]. TSLP is produced mainly by cancer‐associated fibroblasts (CAFs) in response to tumour‐derived cytokines and promotes Th2 polarisation via DCs, facilitating tumour progression [110]. Although both ILC2s and TSLP are individually implicated in pancreatic cancer immunology [110, 111], a direct link between TSLP and ILC2s has yet to be demonstrated but would appear likely.

Despite the extensive data that ILC2s support a more suppressive TME, ILC2s can also exert anti‐tumour effects in specific contexts. ILC2‐effector cytokines drive eosinophilia, and in several tumour types, this is associated with significantly better prognosis [112, 113, 114]. Utilising models of melanoma, ILC2‐mediated eosinophil recruitment was shown to improve tumour control [114]. IL‐33‐driven expansion of ILC2s also appears relevant for effective anti‐tumour responses in hepatocellular carcinoma (HCC), where patients with elevated IL‐33 levels and favourable ILC2 to ILC1 ratios exhibit improved clinical outcomes [115]. In PDAC, an IL‐33:ILC2 axis appears to orchestrate superior CD8 T cell responses, potentially via improved recruitment of CD103+ DCs into the tumour [83].

There is also evidence that direct ILC2:CD8 T cell crosstalk enhances the anti‐tumour response. IL‐33‐driven upregulation of OX40L by ILC2 has been linked to enhanced CD8^+^ T cell activation and better control of melanoma [116]. Furthermore, ILC2s have been proposed to directly cross‐present antigens to CD8 T cells [117]. Although cDC1 are widely considered to be the most proficient cells at cross‐presentation [118], recent studies indicate that ILC2 can also perform this function in vitro. Direct evidence for this in vivo is more limited; however, it does suggest new potential mechanisms through which ILC2 may influence the CD8 T cell response. Where such crosstalk occurs is also of relevance—for cDC1, cross‐presentation is critical within draining lymphoid tissue following egress of activated cells from the tumour. Finally, there is recent evidence that ILC2 can fundamentally shift the immune cell composition of tumours. Within PDAC, IL‐33 has also been shown to drive tertiary lymphoid structure (TLS) formation through expression of lymphotoxin by activated ILC2 [75]. Although the precise roles of TLS in anti‐tumour immunity are currently being dissected, these aggregates are associated with better responses in many cancer types and likely foster superior local effector responses. The ability of some ILCs to phenocopy the embryonic role of LTi cells is a striking discovery [83].

**Summary/Perspective**: As critical mediators of Type 2 cytokine production, ILC2s underpin multiple immunosuppressive mechanisms in the TME that impede effector T cells and facilitate tumour growth. However, in specific contexts, these pathways can also contribute to tumour control. This dualistic nature of ILC2 responses renders them compelling yet challenging targets for cancer immunotherapy, underscoring the need for a nuanced understanding of their context‐specific functions to effectively harness their therapeutic potential. Of note, it is evident that murine and human ILC2s exhibit key functional differences, including, for human ILC2s, the ability to lyse tumour cells via granzyme B [119]. Given the ability to dramatically expand ILC2 numbers ex vivo, this provides an exciting potential cellular therapy and highlights novel potential approaches to exploit ILC biology in enhancing cancer treatments.

***ILC3s***: ILC3s exert their influence through crosstalk with immune but also non‐immune cells, particularly epithelial and stromal elements, shaping the tissue environment in which tumours initiate and evolve [120, 121, 122]. ILC3s are key producers of IL‐22, and multiple studies implicate this pathway in promoting tumour growth [123, 124, 125, 126]. In a model of bacterial‐driven colitis‐associated cancer, the production of IL‐22 is crucial for sustaining CRC, activating the STAT3 signalling pathway in epithelial cells, thereby enhancing proliferation and supporting tumour growth [123]. Furthermore, using the AOM‐DSS model, myeloid: ILC3 cross‐talk through IL‐1β‐induced stimulation of IL‐22 production was found to promote tumour development [124]. This tumour‐promoting function of IL‐22 through STAT3 activation has also been demonstrated in HCC and NSCLC [125, 126]. In a model of HCC, the natural cytotoxicity receptor negative (NCR^−^) ILC3 subset was reported to be the initial responder to IL‐23 signalling during tumour development [127]. These cells rapidly produced IL‐17, a cytokine implicated in the suppression of CD8⁺ T cell proliferation and survival, in response to IL‐23 and expanded in vivo under its influence [128]. Functional relevance was demonstrated through adoptive transfer experiments, where IL‐23 expanded NCR– ILC3s significantly accelerated tumour growth by providing an IL‐17‐enriched immunosuppressive TME [127]. ILC3 production of granulocyte‐macrophage colony‐stimulating factor (GM‐CSF) has also been implicated in sustaining an immunosuppressive TME through recruitment and differentiation of Tregs and TAMs [129, 130, 131]. Finally, ILC3s have been implicated in breast cancer metastasis; receptor orphan receptor (ROR)γt⁺ ILC3s were shown to promote cancer cell dissemination through promoting RANKL expression in stromal cells [132].

However, under certain conditions, ILC3s also contribute to anti‐tumour capabilities. In response to IL‐1β and CCL20 following cisplatin chemotherapy, ILC3s were found to secrete CXCL10, recruiting CD4⁺ and CD8⁺ T cells into the TME, enhancing the efficacy of checkpoint blockade [121]. Dysregulation of the local ILC3 compartment has been identified in patients with CRC, with a reduced ILC3 compartment and expanded ILC1 population [133]. Utilising different mouse models of CRC, ILC3: T cell interactions were demonstrated to play a critical role in supporting tumour control. In addition, IL‐12‐activated NCR^+^ ILC3s have been shown to promote tumour rejection, not through cytotoxicity or IFNγ production, but by inducing expression of adhesion molecules like VCAM‐1 and ICAM‐1 on tumour vasculature, enhancing immune cell infiltration [134]. Blood‐derived human ILC3s have also been reported to directly kill tumour cells, such as those in HCC and melanoma, through TRAIL‐mediated cytotoxicity, suggesting that ILC3s may function as early local sentinels in tissue, responding to malignant transformation or metastasis [135]. The activity of ILC3s is also influenced by microbial metabolites; in HCC, IL‐17A production by ILC3s was shown to increase in response to dysbiosis marked by reduced *Lactobacillus reuteri*, whereas acetate supplementation or faecal transplantation suppressed IL‐17A through HDAC inhibition and Sox13 acetylation, delaying tumour progression and sensitising tumours to PD‐1 blockade [136].

**Summary/Perspective**: ILC3s appear to possess the broadest range of signals to which they can respond and then influence a multitude of other cell types. Among these mechanisms, multiple examples of both pro‐ and anti‐tumourigenic effects have been described.

## 5 Some Current Challenges to Understanding ILCs Roles in Cancer

Several interconnected challenges still impede our understanding of the different roles played by ILCs in cancer [4, 79]. Principally, demonstrating ILC‐specific roles in the context of an otherwise intact immune system remains a key issue. Since the discovery of distinct ILC subsets, the field has struggled to develop in vivo tools that allow for their specific deletion or efficient targeting of molecules expressed by these populations. Such models remain the cornerstone of fully interrogating immune responses. Currently, the most sophisticated genetic models are available for ILC2s [137, 138, 139], facilitating more precise characterisation of their roles. In contrast, models to demonstrate subset‐specific ILC3 or ILC1 roles are lacking.

A further complication arises from the technical challenges in distinguishing ILC1s from NK cells, due to their overlapping phenotypic markers and functional roles. This complicates the interpretation of experimental data and the definitive assignment of functions specifically to ILC1s. Moreover, considerable redundancy exists in the transcriptional and surface marker profiles among ILC subsets, making it difficult to resolve their discrete identities, especially within inflamed or tumour tissue [140, 141]. Single‐cell RNA sequencing (scRNA‐seq) has revealed substantial heterogeneity within ILC populations, suggesting that current classification schemes may not fully encompass their functional diversity [141, 142]. With the current extensive use of scRNA‐seq analyses, transcriptomic insights are frequently not complemented by protein‐level or in vivo validation, limiting their potential functional relevance and translational applicability [4, 142]. Considering human ILCs, robust in vitro models that enable analysis of cellular interactions within tissue remain a work in progress. Critically, unique functions attributable to specific ILC subsets or states may not exist; instead, a layered framework of overlapping cellular roles could provide contingency mechanisms for aberrant responses.

The plasticity of ILCs, which allows them to transition between phenotypes in response to environmental cues [79, 140], may further challenge the study of specific subsets. This becomes particularly relevant in human tissues where longitudinal studies and genetic fate‐mapping approaches are limited. With this topic being extensively reviewed elsewhere [79, 114, 143, 144, 145], we have only briefly touched upon it here. The stability and reversibility of these plastic states, particularly in human cancers, remain poorly understood and impede therapeutic targeting of individual ILC subsets [79, 141].

ILCs are frequently characterised as early and rapid responders, and the pathways that initiate their functions have been mapped out in detail. However, how ILC functions are regulated once initiated is less well understood. T cell exhaustion is a defining feature of chronic pathologies, including cancer. Loss or restriction of effector function in ILCs remains far less defined. Recent studies have identified the expression of immune checkpoint molecules such as PD‐1, TIGIT and CTLA‐4 on ILC subsets [88, 146, 147, 148], suggesting that these cells are subject to inhibitory regulation within the TME. For example, in the context of colitis, CTLA‐4 is expressed on ILC1s and ILC3s, where it plays a role in restraining innate immune activation [147]. Highly activated ILC2s have been shown to express TIGIT, KLRG1 and PD‐1 and are characterised by increased IL‐10 secretion and reduced IL‐5 and IL‐13 expression, suggestive of a regulatory or exhausted phenotype [148]. Notably, interactions between these ‘exhausted’ ILC2s and T cells can promote GATA3 upregulation and drive Th2 polarisation [146]. There is also evidence that ILC1s can become less functional over time in tumours [149]. In these settings, ILC1s exhibit elevated levels of inhibitory molecules like PD‐1 and TRAIL, mirroring aspects of the exhaustion phenotype of T cells [149]. Although the overall number of ILC1s increases, their ability to produce IFNγ is diminished, reflecting a loss of their anti‐tumour capabilities and enabling tumours to evade immune destruction [149, 150]. However, it remains unclear whether the expression of inhibitory receptors on ILCs is constitutive or really induced by the TME. T cell exhaustion is underpinned by distinct transcriptional and epigenetic regulation, and inhibitory receptor expression is just one facet of this cellular state [151]. The molecular pathways governing ILC ‘exhaustion’, if this is a real cellular state, and then its reversibility remain to be properly defined. Determining how ILC functions can be curtailed may facilitate the identification of novel immunotherapeutic strategies to manipulate anti‐tumour responses.

## 6 Conclusions

Building on their roles in orchestrating tissue homeostasis, ILCs have emerged as key cells able to influence the cellular flavour of the TME and help determine the outcome of anti‐tumour responses. Their functional versatility, shaped by the cytokine milieu, tumour type and disease stage, enables ILCs to act as both guardians and collaborators in cancer progression [14, 15]. Group 1 ILCs, including NK cells and ILC1s, are integral to early anti‐tumour responses, but their roles can shift towards exhaustion or even pro‐tumour phenotypes as malignancy advances, reflecting the profound context‐dependence of their activity [36, 77, 78, 82]. Similarly, ILC3s exhibit substantial plasticity, with the potential to transdifferentiate into regulatory or pro‐tumour subsets, complicating mechanistic understanding and therapeutic targeting [52, 109, 152]. The basic cytokine profile of ILC2s and their rapid response to key alarmins typically drive effects that are ultimately pro‐tumourigenic, but as discussed above, tissue context remains important.

The unique properties of ILCs position them as promising, though challenging, targets for cancer immunotherapy. As highlighted above, their potential as cellular therapies is also now emerging. However, despite significant advances, our comprehension of ILC biology in cancer remains constrained by several technical and conceptual challenges. A nuanced understanding of their developmental trajectories, plasticity and interactions within the TME remains essential for harnessing their therapeutic potential. Future research must prioritise the development of refined tools and integrative models to unravel the complexities of ILC function, ultimately informing strategies that can tip the balance towards effective anti‐tumour immunity.

## Conflicts of Interest

The authors declare no conflicts of interest.

## Peer Review

The peer review history for this article is available at https://publons.com/publon/10.1002/eji.70041.

## Data Availability

Data sharing is not applicable to this article as no datasets were generated or analysed during the current study.
